# Dominance of non-Saccharomyces yeasts in artisanal mezcal fermentations

**DOI:** 10.1099/mic.0.001584

**Published:** 2025-10-24

**Authors:** René Quezada, Manuel Kirchmayr, Melchor Arellano-Plaza, J. Alejandro Morales, John P. Morrissey, Anne Gschaedler

**Affiliations:** 1 Department of Industrial Biotechnology, CIATEJ, 1227 Camino Arenero, El Bajío, Zapopan,Jalisco, 45019, Mexico; 2 Department of Computer Science, CUCEI-UdeG, 1421 Marcelino García Barragán, Olímpica, Jalisco 44430, Mexico; 3 SUSFERM Fermentation Science Centre, School of Microbiology, University College Cork, T12 K8AF, Cork, Ireland

**Keywords:** agave, *Lactobacillus*, metataxonomics, mezcal, non-conventional yeast

## Abstract

Mezcal is a spirit obtained from the fermentation and distillation of juices obtained from different agave species. It is one of the distilled beverages with great sociocultural value in different regions of Mexico, and in recent years, it has also gained great economic importance. It is known to present differences in its flavour, thanks to the richness of compounds incorporated within the spirit, which vary according to the agave species used, the microbial population present and the processes involved in its manufacture. This variety reflects the richness of local traditions and the craftsmanship behind its production. The main objective of the present work was to explore parameters that could impact fungal and bacterial diversity. The microbiome of bacteria and yeasts present in fermentations in the same distillery, in two different years and with three different agave species was investigated by metataxonomic analysis obtained from the sequencing of regions V3–V4 for bacteria and ITS1 for yeasts. The results showed that the dominant fungal genera in the fermentations correspond to non-*Saccharomyces* yeasts (*Hanseniaspora, Pichia* and *Zygosaccharomyces*). A major finding was that *Saccharomyces* was not the dominant yeast in any of the 15 fermentations characterized. The dominant bacteria belong to the groups of lactic acid bacteria and acetic acid bacteria. The statistical analysis of the alpha and beta diversities shows that the main statistical differences are seen in the year of fermentation rather than in the species of agave used. Finally, the microbial consortium was composed of the same genera during the different fermentations studied; the fundamental difference was the dominant genus in each fermentation.

## Data Summary

The metataxonomic data and sequences generated in this project were deposited in the NCBI repository under BioProject PRJNA1178143, and a data summary of sequencing information can be found in Table S2. https://dataview.ncbi.nlm.nih.gov/object/PRJNA1178143?reviewer=etrqlt9r7pnp3vk34gj8q5ceu5.

## Introduction

Mezcal, a traditional Mexican alcoholic beverage, is produced through the distillation of fermented juices from different agave species. It is the second most economically significant agave-derived spirit in Mexico, after tequila. Mezcal production has increased significantly, from 980,000 litres in 2011 to 14.16 million litres in 2022 [1,2]. The majority of mezcal production is in Oaxaca state, but the Appellation of Origin protects the production in Durango, Guerrero, San Luis Potosi, Zacatecas and some municipalities of Aguascalientes, Guanajuato, Estado de México, Michoacán, Puebla, Sinaloa and Tamaulipas [2,3]. This increase in production volume has led to a greater demand for raw materials and improved manufacturing process efficiency.

Mezcal production is an artisanal process using over 50 different agave species, varying across different states in the Appellation of Origin [3,6]. These artisanal fermentations reveal an interesting but at the same time complex process due to the variability of practices. In most cases, in the first part of the fermentation (after milling), the crushed agave stems are placed inside the fermentation vat and left for a few days, which gives rise to a semisolid fermentation by autochthonous micro-organisms. With this practice, the fermentation vats undergo a slight increase in temperature, with which the *maestro mezcalero* (master distiller) decides the moment to add water to the fibre, passing to a semiliquid fermentation. This practice differs from standardized processes in tequila fermentations, as it is based on empirical knowledge and experience.

The growing demand for mezcal puts many wild agave species at risk of disappearing and may compromise artisanal qualities due to industrialization. Using commercial starters, for example, can threaten the unique traits contributed by native microflora. Fermentations with agave juice contain diverse micro-organisms (yeasts and bacteria), which are responsible for the unique organoleptic characteristics of each beverage. These include yeasts like *Candida ethanolica*, *Hanseniaspora guillermondii*, *Kluyveromyces marxianus*, *Meyerozyma*, *Metarhizium*, *Pichia kluyveri*, *Saccharomyces cerevisiae*, *Torulaspora delbrueckii* and *Yarrowia* and bacteria such as *Acetobacter* spp*.*, *Lactobacillus* spp., *Leuconostoc* spp. and *Weissella* spp. [3,7]. This microbial diversity likely explains the variations in volatile compounds between batches.

Micro-organisms in mezcal fermentation, including yeasts and bacteria, have been identified using both culture-dependent and culture-independent methods like Polymerase Chain Reaction - Restriction Fragment Length Polymorphism (PCR-RFLP), PCR-denaturing gradient gel electrophoresis (DGGE) and sequencing of the large subunit 26S rRNA gene [8,12]. Identification techniques have evolved with technology. In 1995, Lachance identified 13 yeast species in *Agave tequilana* fermentations using culture-based and morphophysiological methods [13]. Later, molecular tools such as PCR-DGGE, PCR-RFLP and high-throughput sequencing revealed greater microbial diversity, including hard-to-culture or low-abundance organisms involved in tequila and mezcal fermentation [7,9, 11, 12, 14]. Culture-independent methods like PCR-DGGE have limitations, including low sensitivity for detecting less abundant micro-organism [15]. Culture-dependent techniques such as PCR-RFLP may also miss low-abundance species or produce restriction patterns that do not match known profiles.

In the state of Oaxaca, yeast identification in mezcal fermentations has also been carried out through culture and subsequent rDNA sequencing [14,16]. In all these studies, the predominant yeast in fermentation was *S. cerevisiae*. In other studies, the presence of lactic acid bacteria (LAB) and acetic acid bacteria (AAB) in fermentations with agave juice was also detected. A total of nine species corresponding to the bacterial genera of *Acetobacter*, *Bacillus*, *Lactobacillus*, *Lacticaseibacillus*, *Limosilactobacillus* and *Weissella* were identified through PCR-DGGE in fermented musts of *Agave duranguensis* in the state of Durango [11,17]. In fermentations with *Agave salmiana*, eight species of the genera *Lentilactobacillus*, *Limosilactobacillus*, *Weissella* and *Zymomonas* were identified [8,17]. In musts from *Agave angustifolia*, *Agave lechuguilla* and *Agave americana* in the state of Tamaulipas, species of the *Bacillus, Levilactobacillus, Agrilactobacillus*, *Lentilactobacillus*, *Lactiplatibacillus*, *Pediococcus* and *Weissella* were identified [18]. Meanwhile, in the state of Oaxaca, a wide diversity of species was observed in musts from *A. angustifolia*, identifying 36 species belonging to 19 genera. The most abundant genera were LAB such as *Lactobacillus, Leuconostoc*, *Oenococcus* and *Weissella*. Other significant groups included AAB such as *Acetobacter*, *Gluconobacter* and *Komagataeibacter*. The presence of *Bacillus* and *Zymomonas* was also detected in the studied fermentations [14,17].

Metataxonomic studies use molecular biology techniques to identify and characterize microbial communities in specific environments. By extracting and analysing DNA, it is possible to detect the full range of micro-organisms present, including less explored groups, greatly expanding our understanding of microbial diversity [19]. This approach has been applied in various fermentation processes, such as Greek table olives [20], Way-a-linah [21], cacao [22], kefir [23], pulque [24,25] or bacterial communities in the case of mezcal [26], to mention a few.

Mixed microbial populations in artisanal fermentations provide a unique approach to fermentation processes not only in beverages but also in foods, through physical and biochemical changes [27]. Different reports have identified the great diversity that exists in these fermentative processes and the great contribution within the fermentations, such as those observed in cacao [22], cocoa and coffee [28], cocoa bean [29], Greek table olives [20], kefir [23], rice wine and soy-fermented products [30], rice-based fermented products [31], Way-a-linah [21], wine fermentations [32,33] or the aforementioned agave-based fermented beverages, among others.

Due to the limited knowledge on microbial diversity in mezcal fermentations and how it varies by agave species, this study aimed to identify the microbial consortia involved in artisanal fermentations using different agave species within the same distillery over 2 years. Samples were collected at different stages of fermentation and analysed using metataxonomic methods. The PCR-DGGE technique was implemented to corroborate the results of the absence of *S. cerevisiae* as the dominant yeast observed in metataxonomic studies, while RT-PCR was used as a tool to estimate the concentration of bacteria and yeasts present in these fermentations.

## Methods

### Sampling

**Fig. 1.**
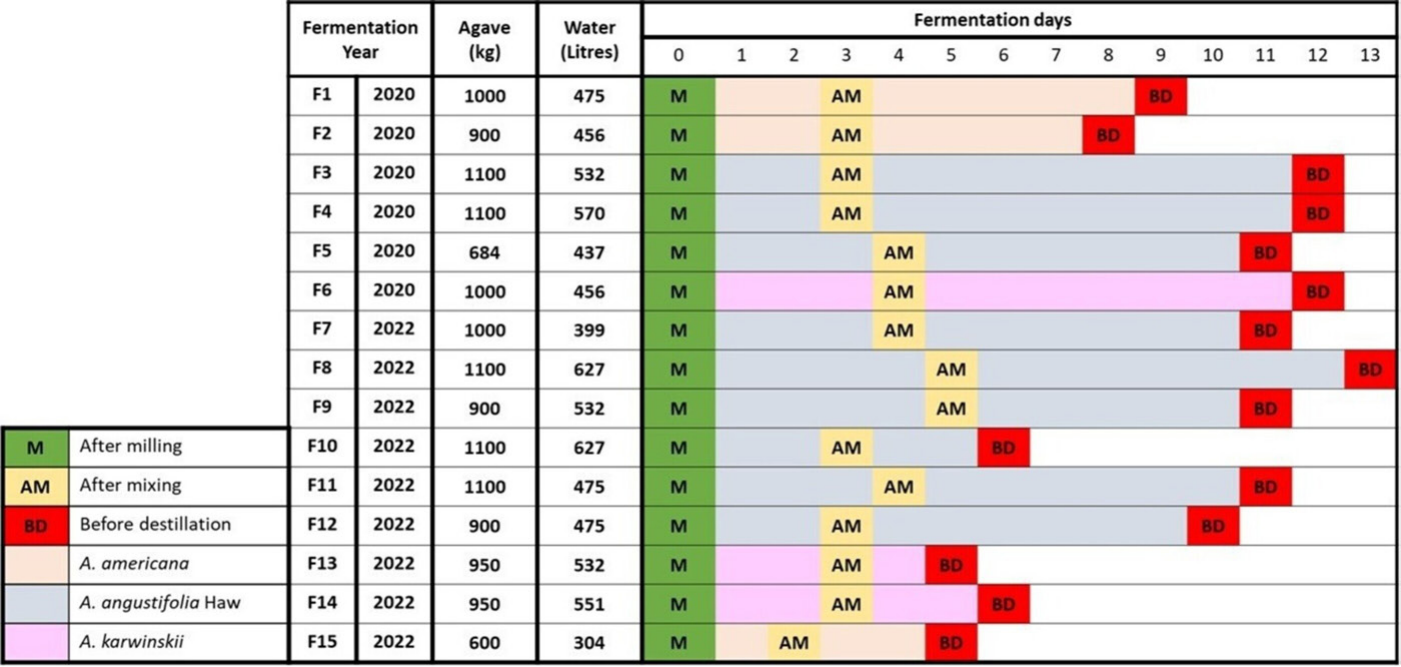
Comparison of fermentation day and agave species in sampling 2020 and 2022. The bars represent the fermentation time. The time at which each of the samples studied was taken is marked.

‘Mezcal de los Angeles, C.C. de R.L.’, an artisanal distillery in the town of Santa Catarina Minas, in the Valles Centrales region of the state of Oaxaca, Mexico, located at 16.7779 N 96.6158 W was the source of the samples for this project. Artisanal fermentation to produce mezcal relies heavily on the empirical knowledge acquired by ‘maestros mezcaleros’ over time. In addition, within the characteristics of this distillery, we can mention its geographical location, as it is separated from any other distillery in the vicinity, the preservation of its spontaneous fermentation processes, due to the fact that it has never used a commercial yeast or inoculum. The different agaves used were harvested on the land owned by the distillery, located in the Valles Centrales region. Subsequently, the process involves the cooking of agave in a pit oven (involving the loss of the micro-organisms present in the raw agave), followed by the milling stage, after which the fibres are placed in the fermentation vat (made of wood). After a waiting period of between 2 and 5 days, depending on the amount of ground agave and following the customs of the ‘maestro mezcalero’, water is added to the fermentation vat, and 1 day later, the vat is mixed. There is no further manipulation until the distillation stage, observed in Fig. S1 (available in the online Supplementary Material). Once the agave is removed, the vat is prepared for the next fermentation, either by superficial washing with water. For our analysis, samples were obtained from 15 independent fermentations in 2 different years, 2020 (F1–F6) and 2022 (F7–F15). Different species of agave, namely, *A. americana*, *A. angustifolia* and *Agave karwinskii*, were used in specific fermentations. For all fermentations, three samples were obtained (~100 ml of each): the first from the liquid fraction generated after milling (M), the second after mixing agave fibre in the vat with water (AM) and the final sample was acquired before the vat underwent distillation (BD). All samples were frozen after collection and until laboratory processing. The precise duration of each fermentation varied as the decision on when the fermentation was complete and ready for distillation was made by the maestro mezcalero. Fig. 1 represents the fermentation times, sampling stages, quantity of agave and water used, as well as the agave species used in each fermentation.

### Quantification of sugar, nitrogen and volatile compounds

The agave set samples were previously microfiltered and placed in 2 ml glass tubes. Quantification of carbohydrates, glycerol and organic acids was performed by HPLC injecting 20 µl in a Waters chromatograph equipped with a refractive index detector (RID Refractive Index Detector) and a BioRad Aminex HPX-87H column (300 mm×7.8 mm, 9 µm). The column was maintained at 50 °C, and 5 mM H_2_SO_4_ was used as mobile phase at a flow rate of 0.5 ml min^−1^ for 30 min. Organic acids were quantified by a wavelength UV detector at 210 nm.

For the determination of organic nitrogen, the technique described by Alcazar in 2011 was used [34]. For the standard solution and preparation of the calibration curve, a solution of l-arginine at 30 mM was used. The Bio-Rad model 680XR microplate reader was used to obtain the absorbance values of the different samples.

The volatile compound determination in the musts was carried out by head-space technique coupled to a GC following the methodology described by Arellano *et al*. [35]. The equipment used for sample injection was the Head Space Hewlett–Packard model HP 7694E coupled to a Hewlett–Packard model HP 6890 GC with a flame ionization detector (FID). Each vial was filled with 2 ml of sample, and after being sealed, the samples were placed on the Head Space carousel and the equipment was programmed with the following conditions: vial temperature 80 °C, loop temperature 110 °C, transfer line temperature 115 °C, vial equilibration 5 min, pressurization time 0.2 min, loop filling time 0.2 min, loop equilibration time 0.5 min, injection time 1 min, and finally injection volume of 1 ml. The HP6890 GC coupled to the FID was programmed as follows: the oven was programmed at 55 °C for 5 min, followed by two temperature increments, the first at 5 °C min^−1^ up to 160 °C and the second at 25 °C/minute up to 220 °C and held at 220 °C for 8 min. The chromatography column was an HP Innowax 60 m×0.32 mm×0.25 µm column. The injector and detector temperatures were at 250 °C. The analysis time was 45 min including head space and GC extraction.

### Metataxonomic analysis

For analysis of the microbial diversity, DNA was extracted from 1 ml of each sample following a previously described procedure by Kirchmayr in 2011 [36], using the commercial GenElute™ Plant Genomic DNA Miniprep Kit from Sigma-Aldrich® and adding 40 µl (200 U) of Lyticase from *Arthrobacter* Sigma-Aldrich® in the extraction process.

Sequencing was conducted using 2×250 paired-end on the Illumina NovaSeq 6000 by Novogene Corporation (USA). For fungal diversity, primers ITS5-1737F and ITS2-2043R corresponding to the ITS1 region [37,39] were used, and for bacterial diversity, the V3–V4 region was amplified using primers 341F and 806R [40,42]. The list of primers and sequences of all methodologies can be found in detail in Table S1.

Microbiome bioinformatics were performed with QIIME 2 2022.2 [43]. Raw sequence data were demultiplexed, denoised, rarefied and quality filtered using the q2‐demux plugin followed by denoising with DADA2 via q2‐dada2 [44]. All operational taxonomic units (OTUs) were aligned with mafft via q2‐alignment [45] and used to construct a phylogeny with fasttree2 via q2‐phylogeny [46]. Taxonomy was assigned to OTUs using the q2‐feature‐classifier and Silva-138-99-nb-classifier [47] database for bacteria and UNITE-ver8-99-classifier [48] for yeast [49,50].

The QIIME 2 platform was used for taxonomic assignment and subsequent data processing (debugging for chimaera removal and sequencing error correction). Derived from 15 fermentations with 3 sampling steps each, 5,098,728 initial sequences were obtained, generating a total of 4,964,486 filtered sequences from 16S amplicon libraries, while from 6,513,007 initial sequences from the ITS amplicon libraries, a total of 5,120,104 sequences were filtered; the details for each sample can be found in Table S3. Only filtered data were used for metataxonomic identification and diversity indices. Alpha and beta diversity was calculated using the QIIME 2 pipeline. Chao1, dominance and Shannon index were determined. Subsequently, an ANOVA statistical analysis was performed in Statgraphics Centurion 19 software to estimate differences between fermentation year, agave species and fermentation stage. In the case of beta diversity, diversity was determined by Bray–Curtis and then a Permutational Analysis of Variance (PERMANOVA) analysis was carried out. Hierarchical clustering was performed and visualized using the R package heatmap3.

### Quantitative evaluation of bacteria and yeasts by quantitative PCR

In each of the samples, relative abundance quantification of populations was performed using quantitative PCR (qPCR). The analysis was conducted using the Rotor-Gene Q Series Software 2.1.0 (Qiagen) detection system, employing a QuantiTect SYBR Green PCR Master Mix (Qiagen); the primers for yeast NL1 and LS2 and for bacteria 338F and 518R were used in accordance with other studies [51,52]. Each reaction contained 10 µl of SYBR Green, 7.2 µl of RNase-free water, 0.4 µl of each primer at 10 µM and 2 µl of DNA. The methodology employed for real-time PCR for yeast was as follows: an initial denaturation step for 15 min at 94 °C, followed by 40 cycles of 30 s at 94 °C for denaturation, 30 s at 52 °C for annealing and 30 s at 72 °C for extension. In the case of bacteria, the methodology used was as follows: an initial denaturation step for 15 min at 94 °C, followed by 40 cycles of 30 s at 94 °C for denaturation, 30 s at 52 °C for annealing and 30 s at 72 °C for extension [53,54]. A standard concentration curve was used to determine the number of total DNA copies, employing *S. cerevisiae* for yeast and *Lactiplantibacillus plantarum* for bacteria. The micro-organisms were inoculated in Yeast Peptone Dextrose (YPD) and de Man, Rogosa, and Sharpe (MRS) medium, respectively, and incubated for a period of 18 h, a cell count was performed in a Neubauer chamber and a plate was made to corroborate viability. An aliquot of 1 ml of the culture medium was obtained and the cell button was frozen. The samples that matched the Petri dish count with the Neubauer chamber were used to create the standard concentration curve.

### DGGE analysis

Genomic DNA extracted from fermentation samples was used for the test samples. In addition, to provide references on the DGGE gels, genomic DNA from a number of yeast species (*Hanseniaspora osmophila*, *P. kluyveri*, *S. cerevisiae*, *T. delbrueckii* and *Zygosaccharomyces bisporus*) was used as a template for PCR reactions. Universal yeast primers were used with modification in the methodology [55, 56]. Both primers were used at a concentration of 10 µM, with forward primer NL1-GC where the non-complementary GC clamp to the target sequences is underlined and reverse primer LS2. The PCR reaction was prepared using 12.5 µl of the GoTaq™ (Promega Corporation) reaction mix, 1.25 µl of each primer, 9 µl of RNase-free water and 1 µl of DNA, resulting in a final reaction volume of 25 µl. The reaction mixtures were processed in a Veriti 96-well thermocycler (Applied Biosystems) programmed under the following conditions: initial denaturation step at 95 °C for 5 min, followed by 30 cycles of denaturation at 95 °C for 1 min, annealing at 52 °C for 1 min, extension at 72 °C for 2 min and a final extension at 72 °C for 10 min. The denaturing gradient concentration used for yeast was 40% for low and 75% for high. Electrophoresis was carried out at 70 V for 16 h in a DCode™ Universal Mutation Detection System (BioRad). The gel was stained with a 0.01% ethidium bromide solution.

For identification of the yeast species represented in the samples, bands were excised from the DGGE gels, recovered in a recovery buffer and subsequently re-amplified using the NL1 and LS2 primers. The obtained products were sequenced through Psomagen Inc. (Rockville, MD, USA). Consensus sequences were identified on the National Center for Biotechnology Information platform through blastn using the Nucleotide Collection (nr/nt) database.

## Results

To study the diversity of fungi and bacteria present during the mezcal fermentation, samples were collected at three stages of agave fermentation in two different years (as described in Methods). As this was an artisanal commercial process, the variations in which agave species were used and what samples were available reflected the practice in the distillery in those years. Fermentations from three different agave species were sampled, nine from *A. angustifolia* and three from both *A. americana* and *A. karwinskii*. DNA was prepared from each sample and metataxonomic analysis was performed using primers specific for fungi or for bacteria, and these datasets were analysed independently. For taxonomic assignment at the genus level, a total of 134 OTUs were established for fungi and 957 OTUs for bacteria, using the UNITE v.8 (99%) database and Silva v.138 (99%) database, respectively.

The concentration of sugars present in the lixiviate (liquid fraction after agave milling) was determined by HPLC, finding a concentration of 70–130 g l^−1^ in *A. americana*, 70–170 g l^−1^ in *A. angustifolia* and 70–150 g l^−1^ in *A. karwinskii*. On the other hand, organic nitrogen concentrations determined by spectrophotometric techniques resulted in concentrations between 0.32 and 0.46 g l^−1^ in *A. americana*, 0.32–0.75 g l^−1^ in *A. angustifolia* and 0.43–0.52 g l^−1^ in *A. karwinskii*. The most abundant volatile compounds analysed by GC-MS were ethyl lactate and ethyl acetate. In the 2020 fermentations, at the end of fermentation, ethyl acetate concentrations were quantified between 400 and 550 mg l^−1^, while in 2022, concentrations ranged from 30 to 110 mg l^−1^. Regarding ethyl lactate, in 2020, concentrations ranged from 200 to 350 mg l^−1^, while in 2022, they were between 30 and 150 mg l^−1^.

### Taxonomic assignment and diversity analysis of fungi

**Fig. 2.**
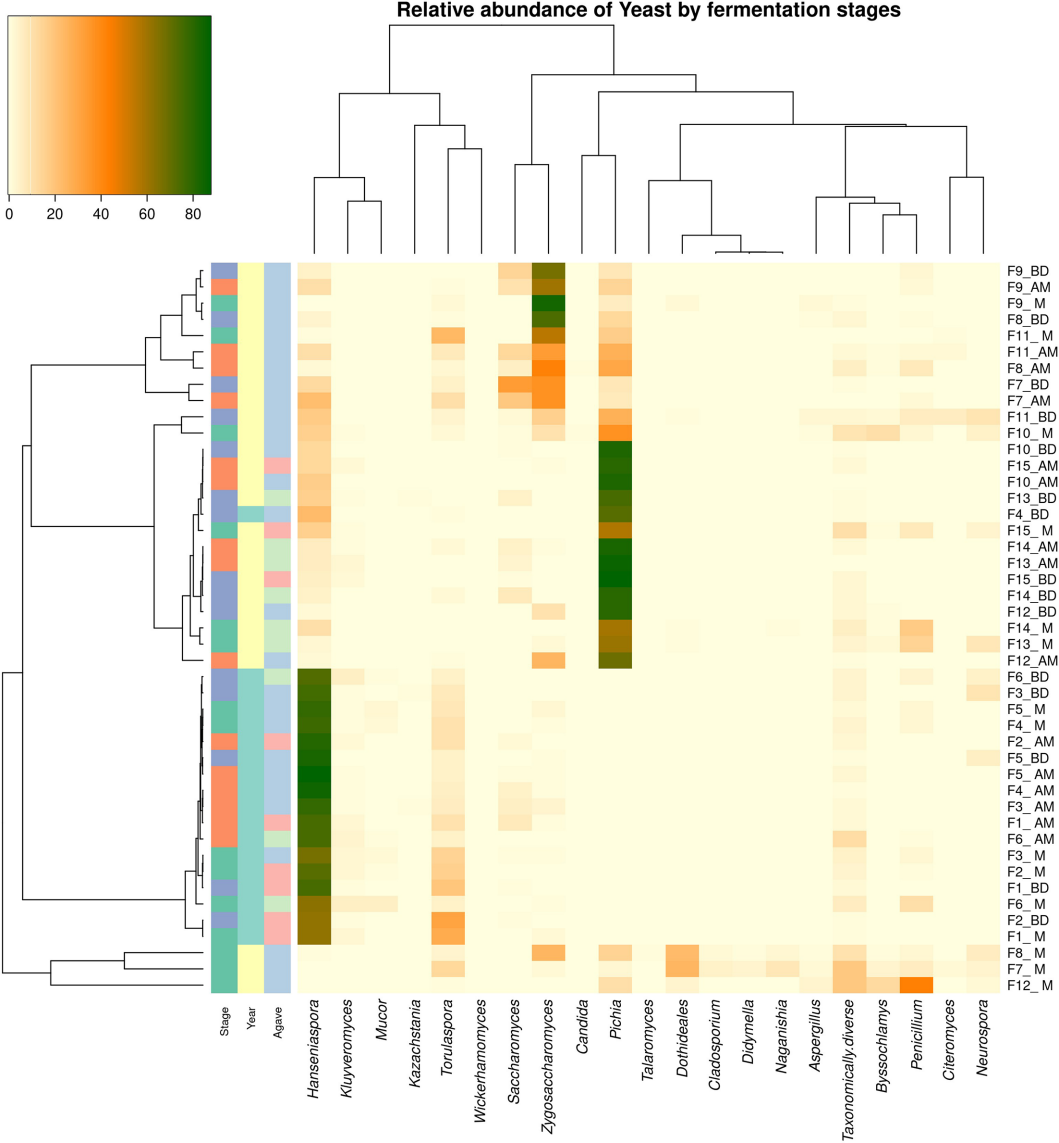
Hierarchical clustering heatmaps of the relative abundance of fungi by fermentation stages. Stage: green, after milling; orange, after mixing; blue, before distillation. Year: green, sampling 2020; yellow, sampling 2022. Agave: red, *A. americana*; blue, *A. angustifolia*; green, *A. karwinskii*.

**Fig. 3.**
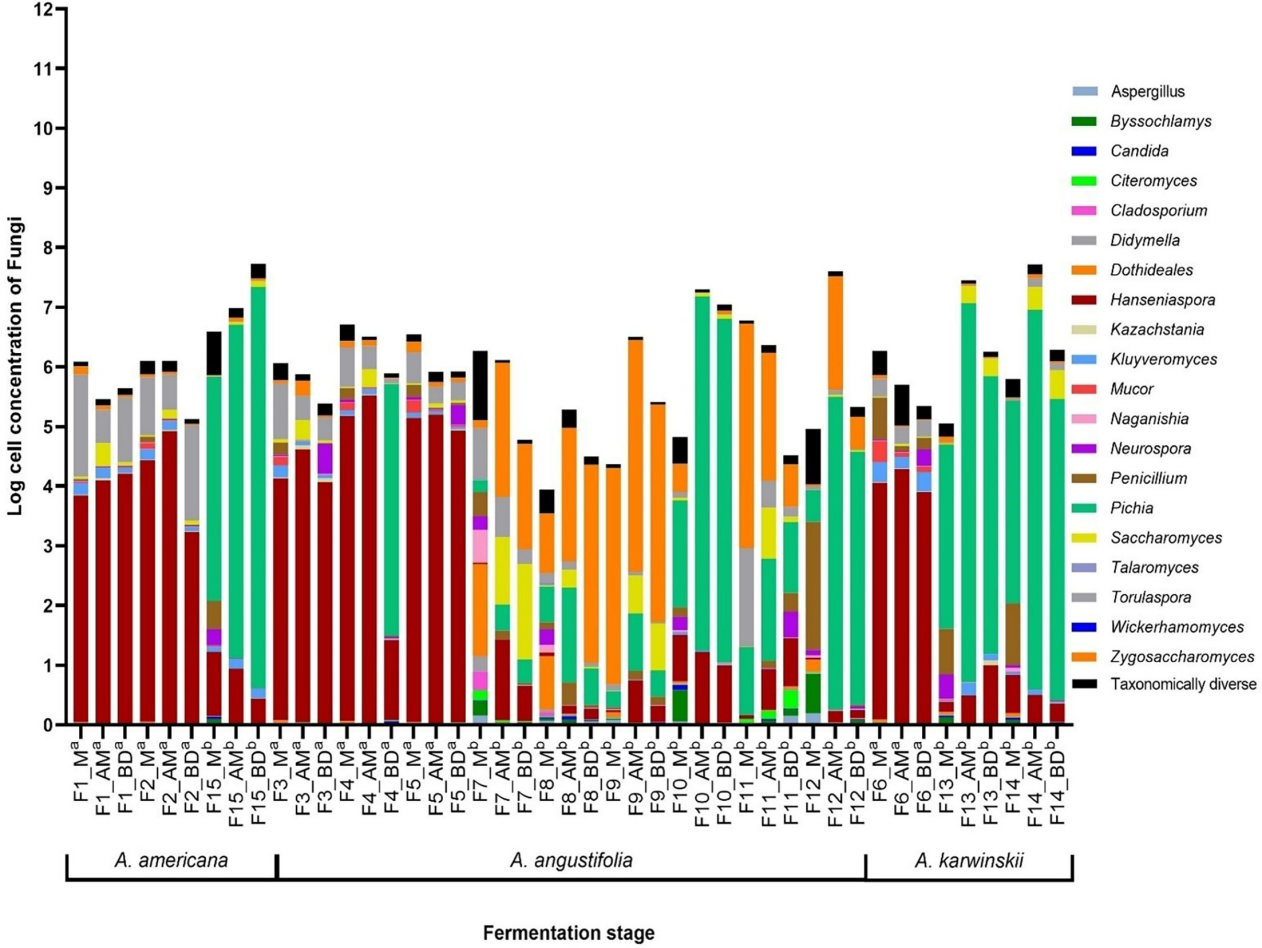
Logarithmic quantification of fungal cells by qPCR. ^a^Sampling 2020. ^b^Sampling 2022. The bars are related to the relative abundance of the main species identified. Concentration was estimated by comparison with a known concentration curve of *S. cerevisiae*.

Considering all samples and after taxonomic assignment, a total of 134 fungal OTUs were obtained. The 20 most abundant OTUs represented between 81 and 99% of the relative abundances found in each process, while the remaining ones were classified as ‘taxonomically diverse’. These data are summarized in a heat map showing those 20 most abundant taxa for the 15 fermentations in their 3 sampling phases in Fig. 2 and shown in Fig. 3 considering the concentrations of total yeast populations. The alpha and beta diversity indices and statistical analysis are presented in Table S4. Data is grouped by year, agave species and sampling stage.

There are several general findings that are readily apparent. First, in all fermentations, a number of different yeast species are present in substantial abundance at each of the three stages of fermentation. Second, while there are some changes over the course of the fermentation, in most cases, all of the yeasts strongly represented at the end of the fermentation were also very evident at the earlier stages and ‘new’ yeasts do not emerge during the fermentation. Third, a small number of genera, namely, *Hanseniaspora*, *Pichia*, *Torulaspora* and *Zygosaccharomyces*, are the dominant taxa and, while *Saccharomyces* is sometimes present, it is never the most abundant taxon in the fermentation. Fourth, the dominant yeast taxa vary between fermentation and are more strongly associated with the year than the agave variety.

A clear trend was for the yeast taxon that was dominant at the first stage (M) to increase both its total numbers and its dominance but not to the complete exclusion of all other taxa by the end of the fermentation (BD) (Fig. 3). There were a few notable exceptions, however. For example, in F4, which was a fermentation with *A. angustifolia* in 2020, *Hanseniaspora* was replaced by *Pichia* as the dominant taxon by the final stage (BD). And in F7 and F11, other *A. angustifolia* fermentations, but this time in 2022, the fermentation remained quite diverse without a single dominant taxon emerging. Interestingly, both of these fermentations were also notable for a substantial drop in the total yeast counts in the BD samples, indicating perhaps some unusual feature of these particular fermentations.

The predominance of different taxa in 2020 and 2022 was striking. In 2020, represented F1 to F6, the dominant taxon in all fermentations was *Hanseniaspora*, with *Torulaspora* also strongly represented. In contrast, in 2022, while *Hanseniaspora* and *Torulaspora* were both well represented, most fermentations were dominated by either *Pichia* (F10, F11, F12, F13, F14 and F15) or *Zygosaccharomyces* (F8, F9 and F10). The surprising finding was that although *Saccharomyces* was detectable at some level in the majority of the fermentations, it achieved its highest level in F7 (33% of the total counts) and was never dominant.

**Fig. 4.**
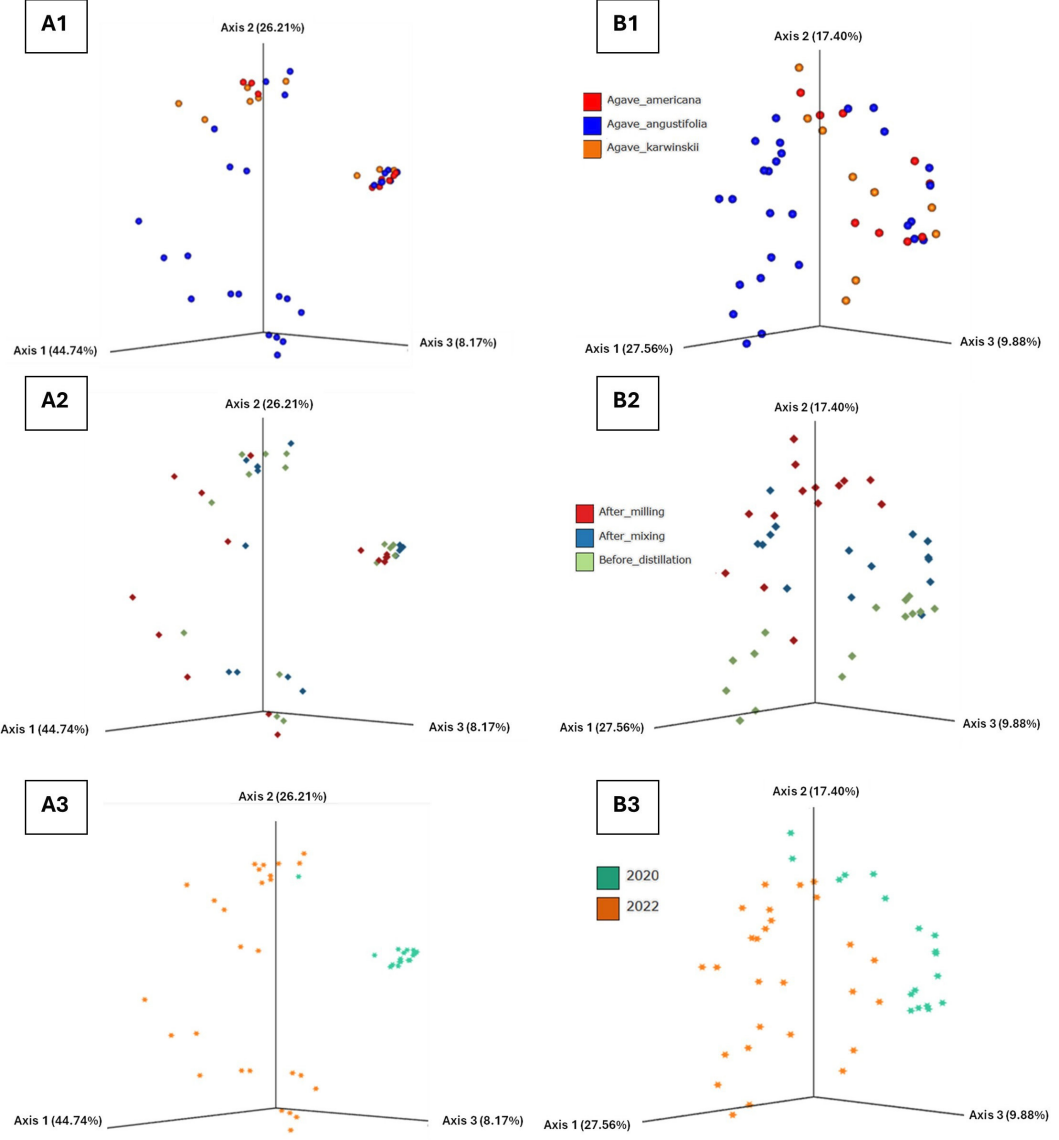
PCoA based on the Bray–Curtis dissimilarity. Panels A1–A3 represent the fungal PCoA with the variable agave species (A1), stage (A2) and year (A3) explaining 79.12% of the total observed variation. Panels B1- B3 represent the bacterial PCoA with the variable agave species (B1), stage (B2) and year (B3) explaining 54.84% of the total variation.

Several different indices were used to quantify the diversity in the samples (Table S3). This allows comparisons to be made throughout fermentation and between different fermentations. The Chao1 index is an estimator of the number of species in a community based on the number of species in the sample. The dominance index used to determine whether a taxon dominates fermentation is the closer the value is to 1. The Shannon index is used to determine the diversity of a community; the higher the value, the more diverse it is. Values near or above 3 indicate high diversity. Taking the diversity indices and based on the results of the ANOVA statistical analysis, some differences were observed in the diversity of micro-organisms in the fermentations based on the year of sampling, agave species and fermentation stage. According to the Chao1 index, the difference between the number of species identified is only significant between fermentation stages, finding a greater difference after milling (stage M=86.47±25.18). The dominance is significant only between fermentation years, finding higher dominance in the 2020 fermentations in each of the fermentation stages. Diversity according to the Shannon index is significant between years and fermentation stages, finding higher diversity again after milling (stage M=2.58±0.84). For beta diversity, the pseudo-*F* values allow measuring the variability between groups in comparison with the variability within groups, so that the higher the value, the greater the variability. Based on the results of the PERMANOVA (Table S4) and principal coordinate analysis (PCoA) in Fig. 4, it is observed that there is only a significant statistical difference taking the year as the study variable. The agave species did not make a statistically significant difference in any case.

### Taxonomic assignment and diversity analysis of bacteria

**Fig. 5.**
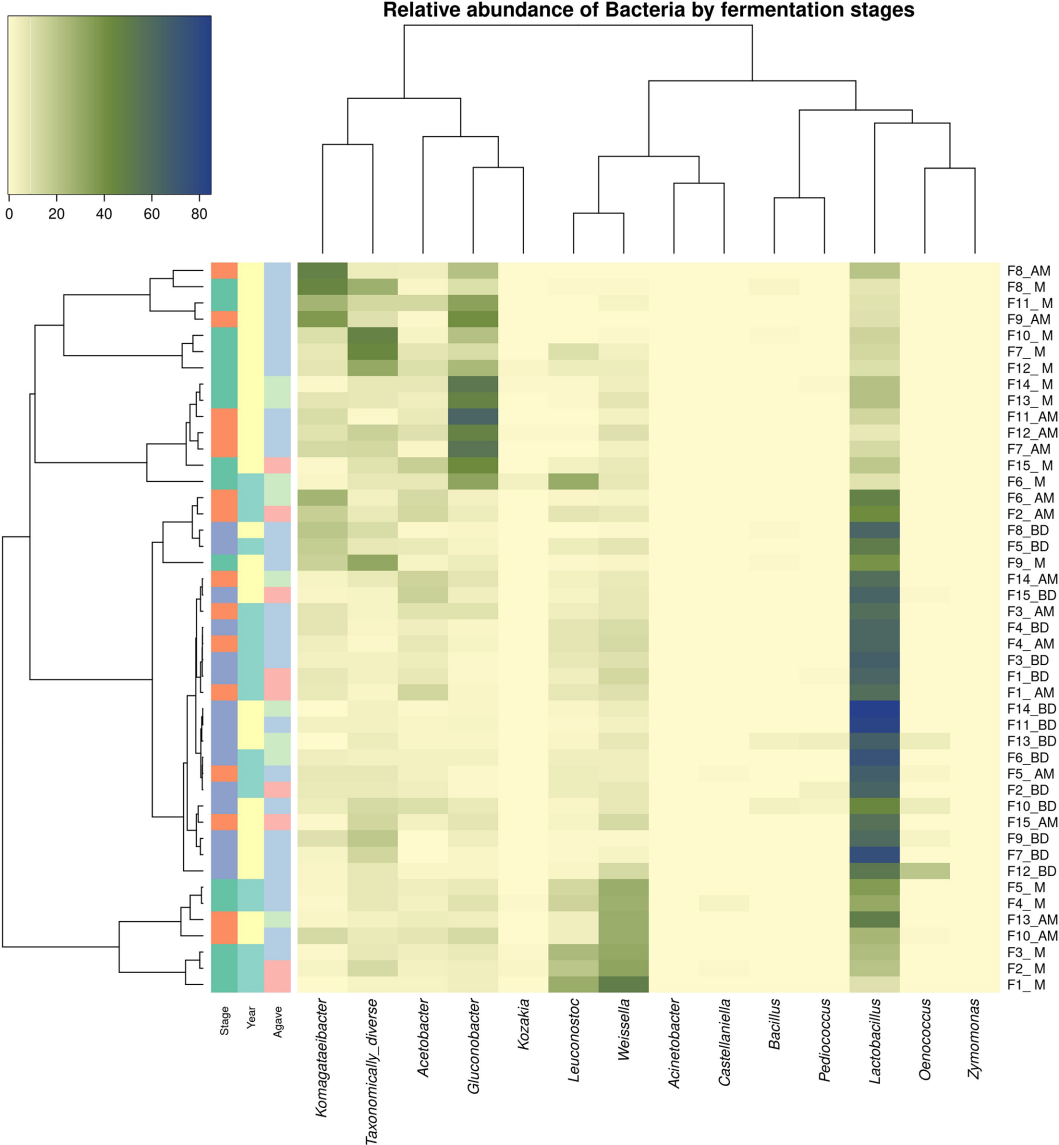
Hierarchical clustering heatmaps of the relative abundance of bacteria by fermentation stages. Stage: green, after milling; orange, after mixing; blue, before distillation. Year: green, sampling 2020; yellow, sampling 2022. Agave: red, *A. americana*; blue, *A. angustifolia*; green, *A. karwinskii*.

**Fig. 6.**
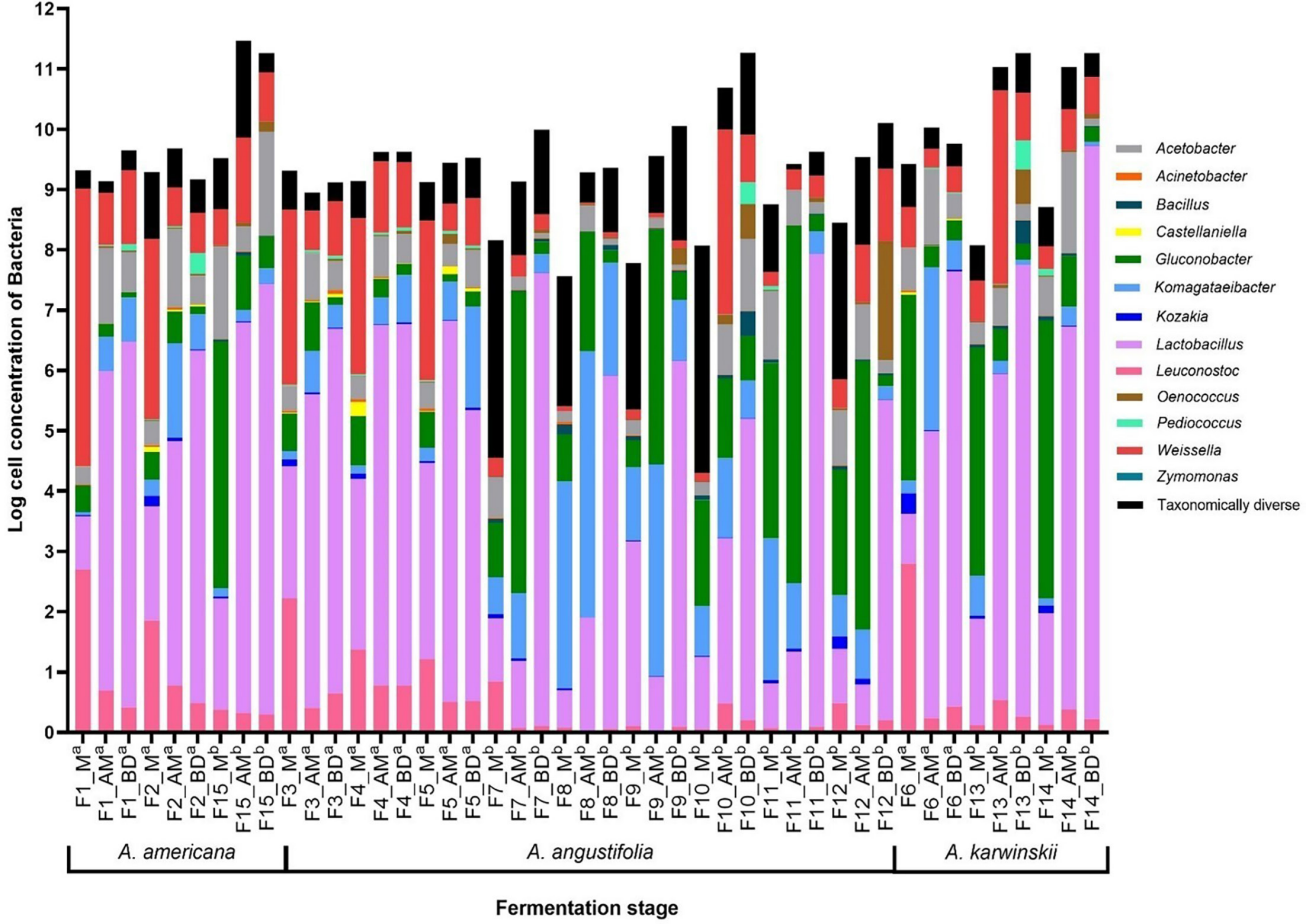
Logarithmic quantification of bacterial cells by qPCR. ^a^Sampling 2020. ^b^Sampling 2022. The bars are related to the relative abundance of the main species identified. Concentration was estimated by comparison with a known concentration curve of *L. plantarum*.

Subsequent to taxonomic assignment, a total of 957 fungal OTUs were obtained, of which between 55 and 99% of the relative abundance are grouped into 13 OTUs, while the remaining were classified as ‘taxonomically diverse’. These data are presented in a heat map showing the 13 most representative taxa for the 15 fermentations in their 3 sampling phases in Fig. 5, and the concentrations of total yeast populations are shown in Fig. 6. The alpha and beta diversity indexes and statistical analysis are presented in Table S5. These results allow us to appreciate the dominant bacterial populations in each sampling, as well as the existence of statistically relevant differences between them.

In all fermentations, several species of LAB and AAB are present in great abundance in each of the three fermentation phases. Although changes occur throughout fermentation, in most cases, all representative bacteria at the end of fermentation are present in the earlier stages, and no new species emerge during fermentation. Third, *Lactobacillus*, *Leuconostoc* and *Weissella* from the LAB group and *Acetobacter*, *Gluconobacter* and *Komagataeibacter* from the AAB group are the dominant taxa. Fourth, the dominant bacterial taxa vary among fermentations and are more strongly correlated with year than with agave variety.

It is important to emphasize that no apparent dominant taxon is seen in the first phase (M) of fermentation, while as the process progresses, the genus *Lactobacillus* increased both in total number and dominance, but not to the complete exclusion of all other taxa at the end of fermentation (BD) (Fig. 5). However, there were some notable exceptions. For example, *Leuconostoc* and *Weissella* genera (LAB) were among the most abundant groups in the M-phase and decreased as fermentation progressed until the end of fermentation (BD). On the other hand, AAB belonging to *Acetobacter, Gluconobacter* and *Komagataeibacter* also decreased in total number as fermentation progressed.

The difference in predominant taxa between 2020 and 2022 was striking. In 2020, represented from F1 to F6, the dominant taxon in all fermentations corresponded to LAB genera. On the contrary, in 2022, corresponding to F7 to F15, the presence of AAB and LAB in similar proportions stands out; however, there is a greater diversity of bacteria in this year. It is important to note that in both cases at the end of fermentation, the dominant taxon was *Lactobacillus*.

Based on diversity indices and the results of the ANOVA and PERMANOVA statistical analysis (Table S4), some differences in the diversity of micro-organisms were observed based on the year of sampling, type of agave and fermentation stage. According to the Chao1 index, the difference between the number of species identified is significant between the years 2020 and 2022, as well as finding a greater variation at the beginning of fermentation (stage M=515.23±265.20). Within the agave species, a significant difference was found in the dominance at the beginning of fermentation, being the fermentations with *A. angustifolia* the ones with the greatest difference (stage M=0.06±0.04). The diversity according to the Shannon index is significant between fermentation years, finding a greater diversity at the beginning of fermentation in the year 2022 (stage M); however, as fermentation progressed in stage AM, this difference is inverted, finding a greater diversity in the year 2020. The beta diversity and PERMANOVA (Table S5) and PCoA in Fig. 6b results demonstrate a statistically significant difference between fermentation years and, in this case, between fermentation stages. However, again, according to this parameter, the agave species does not present a significant statistical difference.

### Confirmation of sequencing results by DGGE

**Fig. 7.**
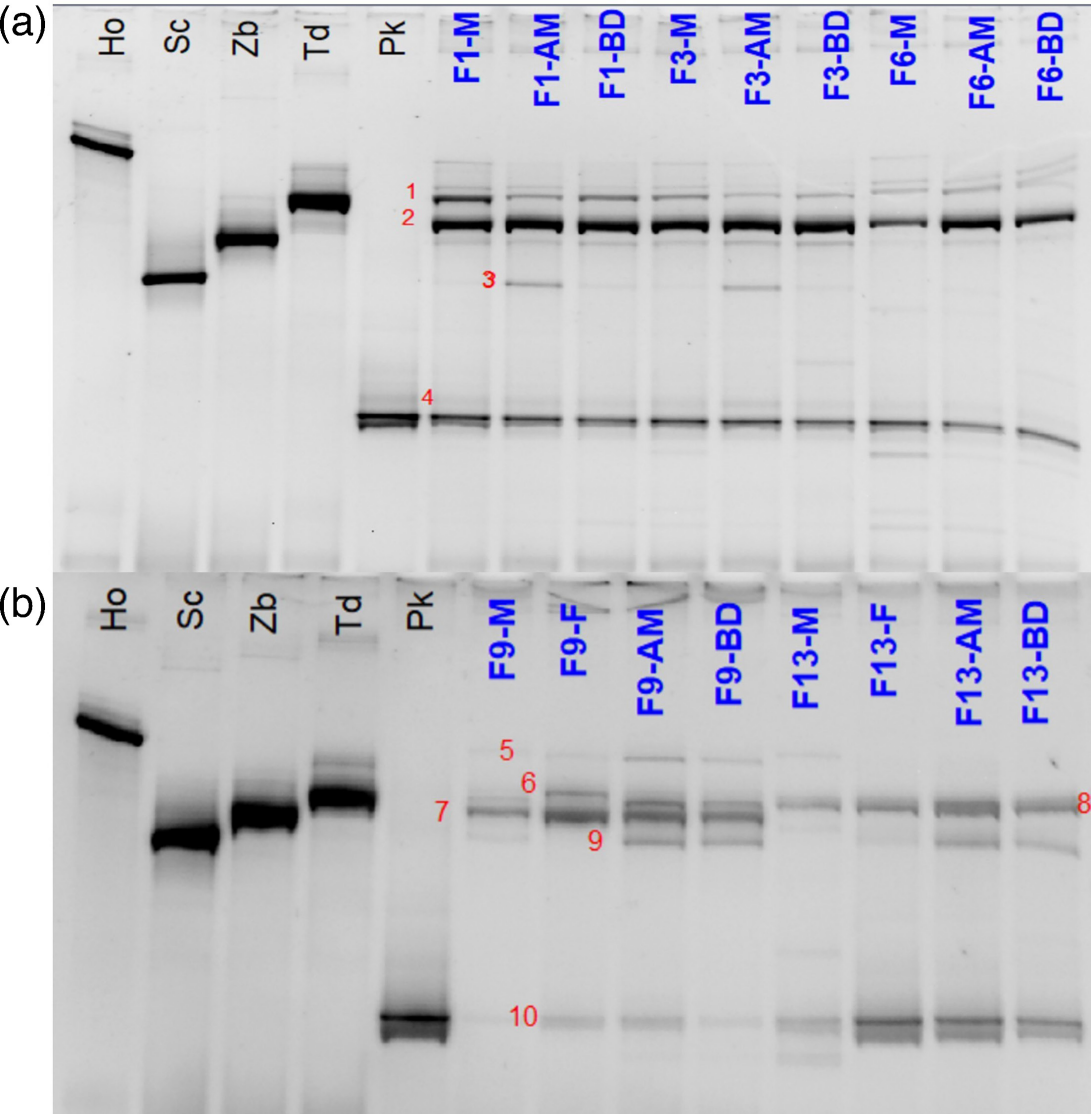
Yeast DGGE. Abbreviations: A, sampling 2020; B, sampling 2022; Ho, *H. osmo*phila control; Sc, *S. cerevisiae* control; Zb, *Z. bisporus* control; Td, *T. delbrueckii* control; Pk, *P. kluyveri* control. Lines F represent profiles of the DNA amplicons obtained from the fermentations analysed. The numbers represent the identified bands: 1, *T delbrueckii*; 2, *Hanseniaspora guilliermondii*; 3, *S. cerevisiae*; 4, *P. kluyveri*; 5, *H. osmophila*; 6, *T*. *delbrueckii*; 7, *Z. bisporus*; 8, *Z. bisporus*; 9, *S. cerevisiae*; 10, *P. kluyveri*.

Some of the findings of the metataxonomic analysis were both striking and unexpected, so it was decided to analyse a subset of the samples using a different method, DGGE (Fig. 7). For this, a portion of the rDNA is amplified and PCR products are separated based on sequence and structure rather than length. Using this method, each band on a denaturing gel represents a species. The particular species can be identified based on comparison to a reference or by excising, reamplifying and sequencing the band.

In each of the three fermentations from 2020 that were analysed, *Hanseniaspora guilliermondii* was the dominant population and both *T. delbrueckii* and *P. kluyveri* were present. *S. cerevisiae* was detected in F1 and F3, though abundance was low and some other low-abundance bands were visible but not identified in several samples. The data generally supported that seen in the metataxonomic identification other than the presence of *Pichia*, which was not detected at all in these samples in the metataxonomic analysis. The data from the three 2022 fermentations also largely corroborate the metataxonomic analysis (Fig. 7b). *Z. bisporus* and *P. kluyveri* are the dominant yeasts present in all three fermentations. The relative abundance estimated in the metataxonomic analysis agrees with that observed in the DGGE gels. This is especially evident for fermentation F9, where *Zygosaccharomyces* was the most dominant population.

### Quantitative evaluation of bacteria and yeasts by qPCR

The cell concentrations of both bacteria and yeast are depicted in Figs3  5, showing yeast concentration oscillates between 3.94 and 6.78 logarithms in the stage M, 5.28 and 7.72 in the stage AM and 4.49 and 6.29 in the stage BD. Bacterial concentration oscillates between 7.5 and 10.95, 8.94 and 11.47 and 9.12 and 11.27, respectively. The bacterial population increases at the end of fermentation (BD), and this phase exhibits the greatest difference between yeast and bacterial concentrations, with the smallest difference observed on average at the beginning of fermentation (M).

Fermentations with *A. americana* and *A. karwinskii* show a higher increase in both yeast and bacterial concentrations in the 2022 samplings compared with 2020. Conversely, fermentations with *A. angustifolia* exhibit the lowest yeast and bacterial concentrations in the sampling 2022. Fermentations F10 (*A. angustifolia*), F13–F14 (*A. karwinskii*) and F15 (*A. americana*) displayed the highest concentrations of both bacteria and yeast, with fermentation times ending between 5 and 6 days. Additionally, it is noted that in other fermentations with *A. angustifolia*, the lowest micro-organism concentrations were observed, and these also had longer fermentation times.

## Discussion

It is important to highlight that there are no previous references on metataxonomic identification involving yeasts and bacteria in mezcal fermentations in Mexico. Therefore, this study is a pioneer in identifying the microbial consortium present in artisanal fermentation processes in the state of Oaxaca, in addition to the fact that these processes, despite being from the same distillery, were carried out with different agave species.

The presence and dominance of *S. cerevisiae* have been reported in several agave fermentations [10,12, 14, 57]; however, the absence of *S. cerevisiae* has also been observed in some fermentations with *A. salmiana* [8]. It is believed that the appearance of different yeast genera in traditional fermentations could be explained by the dispersing action of insects, so it is thought that *S. cerevisiae* could have been introduced by dispersal and, once installed, become a permanent microbial resident in the mezcal production process [10]. Agave juice presents a challenging fermentation environment due to its lower nitrogen concentration, as well as the presence of antimicrobial compounds such as saponins and furfurans, which may suggest that yeast interactions in agave fermentation differ from those of other fermentation processes [58]. In addition to the factors mentioned above, the fact that this distillery is kept separate from other distilleries, the conservation and in theory isolation of its fermentation processes and reports of *Saccharomyces* distribution and displacement to non-native sites [59,61], could be an explanation for the non-dominance of this yeast within this distillery.

Previous studies have reported that yeasts such as *Hanseniaspora* and *Pichia* sp. can esterify alcohols such as ethanol, geraniol, isoamyl alcohol and 2-phenylethanol, resulting in increased concentrations of their respective esters [8,58, 62, 63], which could explain the increase in ethyl lactate and ethyl acetate in the 2020 fermentations due to the dominance of yeasts of the genus *Hanseniaspora*, which did not occur in 2022. In agave spirits such as mezcal, the presence and concentration of volatile compounds are crucial for the quality of the beverage. Although their presence has been reported in fermentations with agave juice, the dominance of non-*Saccharomyces* in these processes is not common. On the other hand, in fermentations with other raw materials, this phenomenon has been observed. For example, the abundance of the *Hanseniaspora* in cocoa fermentations is common due to its tolerance to low pH [22]. Studies on the microbial flora associated with Way-a-linah (an alcoholic beverage generated from fermented sap of *Eucalyptus gunnii*) and tuba (fermented beverage from *Cocos nucifera*) found dominant species of *Hanseniaspora*, followed by *Lachancea* and *Candida*, mainly attributed to environmental and anthropogenic factors that are determinants in the composition of microbial communities found in the soil or associated with plants and fruits [21]. In grape fermentations, a greater diversity of non-*Saccharomyces* yeasts has been found, primarily due to the lack of raw material cooking. In these fermentations, some species of the genera *Candida*, *Debaryomyces*, *Hanseniaspora*, *Issatchenkia*, *Kluyveromyces*, *Kloeckera* and *Torulaspora* have been identified [64,66]. However, these are not dominant within the fermentation.

Studies on bacteria present in the fermentation of agave musts are even scarcer than those on yeasts. Their role within fermentation is primarily described in the production of organic acids and some precursors for the generation of elements such as esters, contributing important organoleptic properties [11,17].

Metataxonomic analyses for the identification of micro-organisms in fermented products are currently an excellent tool for identifying minority groups and potentially non-cultivable organisms. For example, micro-organisms present in aguamiel fermentations obtained from *A. salmiana* used in pulque production have been successfully identified. This was achieved by amplifying V3–V4 fragments of the 16S rRNA gene for bacteria and the ITS region for yeasts, allowing the identification of OTUs with higher bacterial abundance such as *Acetobacter*, *Gluconobacter*, *Lactobacillus*, *Lactococcus*, *Leuconostoc*, *Obesumbacterium*, *Weissella* and *Zymomonas*. Fungal OTUs included *Hanseniaspora*, *Kazachstania*, *Kluyveromyces* and *Saccharomyces*. In this report, *Zymomonas* was found to be dominant among bacteria, while *Saccharomyces* dominated among yeasts [25]. There is an observed difference in the relative abundances obtained in this mezcal study, as non-*Saccharomyces* genera dominate among yeasts and the LAB group among bacteria.

The diversity indices allowed observing changes in the evolution of microbial consortia present in these fermentation processes. The diversity indexes allowed observing changes in the evolution of the microbial consortia present in these fermentation processes. Observing that the type of agave does not play a significant role in the microbial population present, the greatest variability was found between fermentation years and above all a greater diversity in the initial stage of fermentation. Compared with this study, lower diversity was observed in pulque than in mezcal [25]. In general, different research on identification and diversity has been used to assess microbial richness and diversity in several fermentations, such as cocoa bean [29], wine fermentations [32,33], cocoa and coffee [28], rice wine and soy fermented products [30] and rice-based fermented products [31], to mention a few.

The qPCR technique has been used to quantify total bacterial and yeast populations in several studies. The use of 16S regions for bacteria and ITS1 for yeast has proven to be useful for this process. Compared with traditional quantification methods, the use of qPCR has shown a good relationship with these. Despite slight variations caused by factors such as DNA extraction or possible variation due to dead cell estimation, it is considered a reliable, efficient and rapid technique [51,54,67]. It can also be an excellent tool to include microbial populations that are difficult to culture or in cases where the sample cannot be cultured on the spot and has to be frozen, for example.

## Conclusions

This study revealed the microbial consortia present in fermentations carried out with three different agave species within the same distillery and across different sampling years. Metataxonomic studies significantly contribute to characterize the microbial populations present during the different fermentation stages. Additionally, it enables the identification of micro-organisms found in small concentrations.

The main finding in this study reveals that the dominant yeast in each stage and fermentation process belongs to non-*Saccharomyces* species in the case of yeasts and to *Lactobacillus* genus in the case of bacteria. Despite occurring in the same distillery, the genera, their dominance and their concentrations vary even within fermentations in the same year. In 2020, the dominant yeast was *Hanseniaspora*, while in 2022, the dominant populations were *Pichia* and *Zygosaccharomyces*. In both years, the dominant bacterial genera corresponded to the LAB group. It was shown that the year had a greater influence on the microbial consortia’s composition than the agave species that was employed.

This is the first metataxonomic study involving the identification of both yeasts and bacteria that has been conducted on mezcal fermentations. It is now necessary to extend the research to broaden microbial characterization and to determine the factors that may have a direct impact on the composition of the microbial consortium. In the future, this knowledge can be applied to understand the role played by consortium members in the generation not only of ethanol, but also of other volatile compounds and components that contribute to the unique organoleptic properties of mezcal.

Knowledge of the micro-organisms present in fermentation not only helps us to improve the fundamental understanding of these biological processes but is also fundamental to understanding the biochemical processes involved and to optimize, control and regulate fermentation conditions. This can lead to the improvement of the fermented products and to the developmment of strategies that make their production more efficient and profitable.

## Supplementary material

10.1099/mic.0.001584 Uncited Supplementary Material 1.

## Abbreviations

AAB: acetic acid bacteria
DGGE: denaturing gradient gel electrophoresis
LAB: lactic acid bacteria
OTUs: operational taxonomic units
PCoA: principal coordinate analysis
PCR-RFLP: Polymerase Chain Reaction - Restriction Fragment Length Polymorphism
PERMANOVA: Permutational Analysis of Variance
qPCR: quantitative PCR
