# Supplementary material for: Dominance of non-Saccharomyces yeasts in artisanal mezcal fermentations
Source: Microbiology (Reading). 2025 Oct 24;171(10):001584. doi: 10.1099/mic.0.001584 (PMC12551765; doi:10.1099/mic.0.001584)
Supplement: Uncited Supplementary Material 1. [file mic-171-01584-s001.pdf]

SUPPLEMENTARY INFORMATION

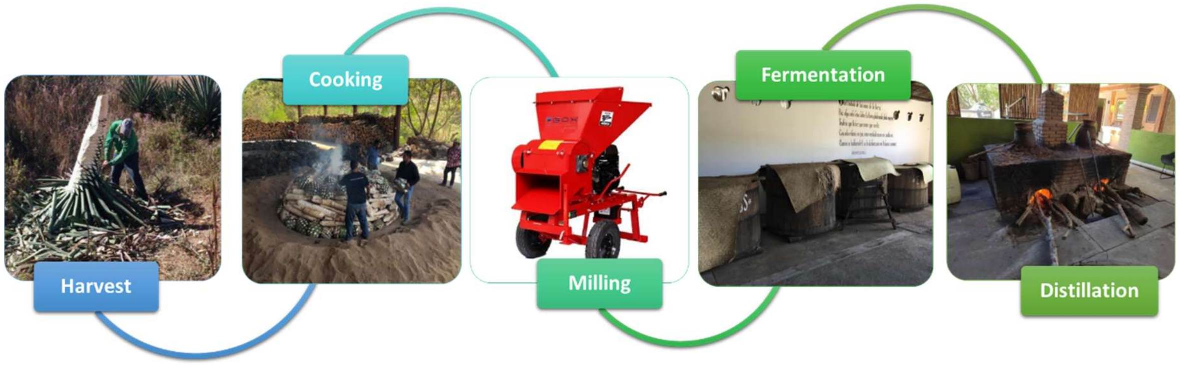

Figure S1. Mezcal production process used in Real Minero Distillery.

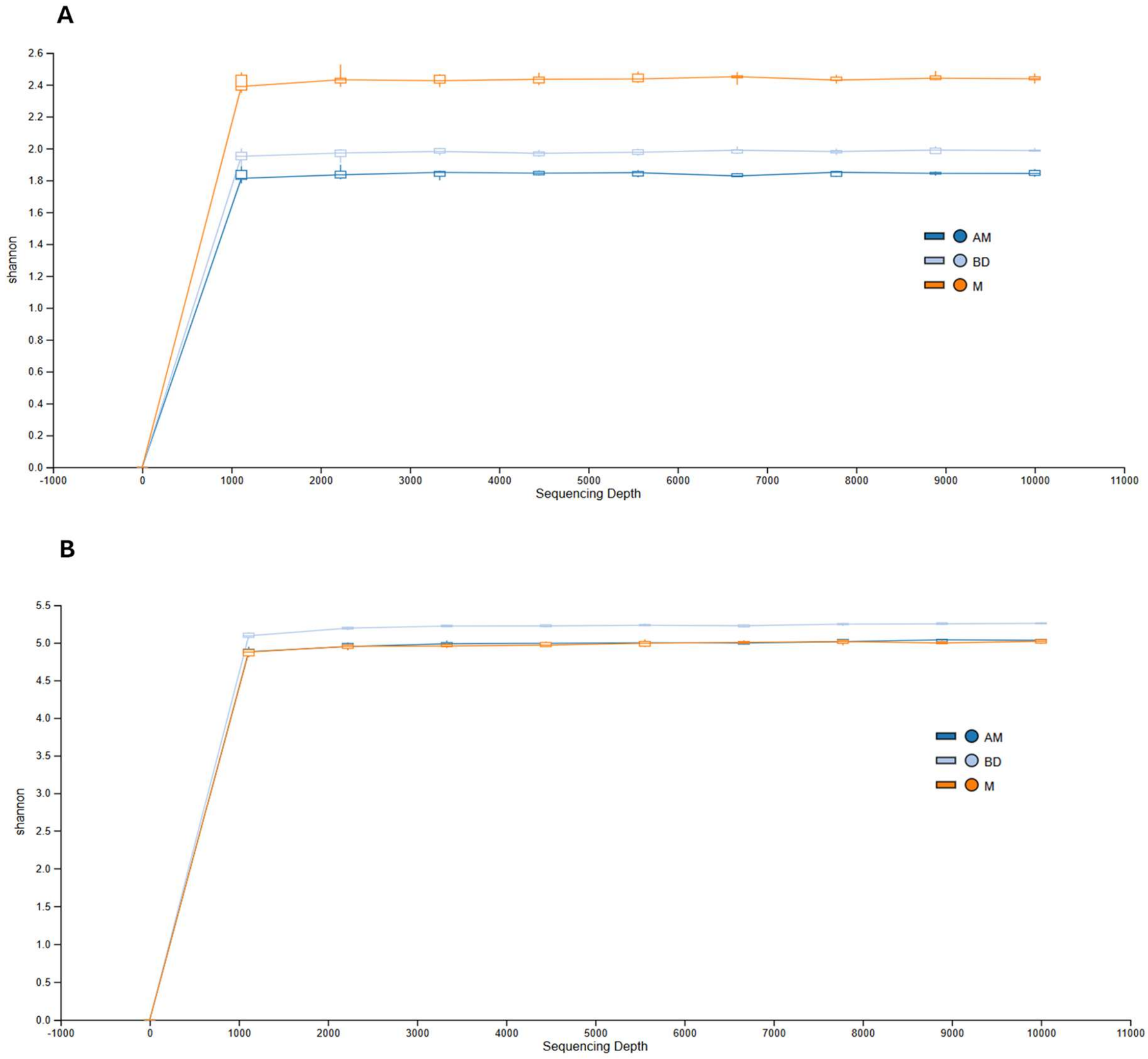

Figure S2. Rarefaction curves presenting the relationship between sequencing depth and the Shannon index using stages of fermentation. **A.** Rarefaction curves for fungi. **B.** Rarefaction curves for bacteria.

Table S1. Sequence of primers used for amplification of the ITS1 region in fungal diversity and the 16S region of bacterial diversity.

Metodology	Microorganism	Primers	Sequence
Metataxonomic analysis	Fungal diversity	ITS5-1737F	GGAAGTAAAAGTCGTAACAAGG
		ITS2-2043R	GCTGCGTTCTTCATCGATGC
	Bacterial diversity	341F	CCTAYGGGRBGCASCAG
		806R	GGACTACNNNGGGTATCTAAT
qPCR	Yeast	NL1	ATATCAATAAGCGGAGGAAAAG
		LS2	ATTCCCAAACAACTCGACTC
	Bacteria	338F	ACTCCTACGGGAGGCAGCAG
		518R	GGACTACNNNGGGTATCTAAT
DGGE	Yeast	NL1-GC	CGCCCGCCGCGCGCGGCGGGCGGGGCGGGGGCC-ATATCAATAAGCGGAGGAAAAG
		LS2	ATTCCCAAACAACTCGACTC

Table S2. Data summary of sequencing information used for metataxonomic analysis.

Accession number	Sample code	Description
SAMN44463287	F1_M_Y	Fermentation_1_After-milling_(Yeast)
SAMN44463288	F1_AM_Y	Fermentation_1_After-mixing_(Yeast)
SAMN44463289	F1_BD_Y	Fermentation_1_Before-distillation_(Yeast)
SAMN44463290	F2_M_Y	Fermentation_2_After-milling_(Yeast)
SAMN44463291	F2_AM_Y	Fermentation_2_After-mixing_(Yeast)
SAMN44463292	F2_BD_Y	Fermentation_2_Before-distillation_(Yeast)
SAMN44463293	F3_M_Y	Fermentation_3_After-milling_(Yeast)
SAMN44463294	F3_AM_Y	Fermentation_3_After-mixing_(Yeast)
SAMN44463295	F3_BD_Y	Fermentation_3_Before-distillation_(Yeast)
SAMN44463296	F4_M_Y	Fermentation_4_After-milling_(Yeast)
SAMN44463297	F4_AM_Y	Fermentation_4_After-mixing_(Yeast)
SAMN44463298	F4_BD_Y	Fermentation_4_Before-distillation_(Yeast)
SAMN44463299	F5_M_Y	Fermentation_5_After-milling_(Yeast)
SAMN44463300	F5_AM_Y	Fermentation_5_After-mixing_(Yeast)
SAMN44463301	F5_BD_Y	Fermentation_5_Before-distillation_(Yeast)
SAMN44463302	F6_M_Y	Fermentation_6_After-milling_(Yeast)
SAMN44463303	F6_AM_Y	Fermentation_6_After-mixing_(Yeast)
SAMN44463304	F6_BD_Y	Fermentation_6_Before-distillation_(Yeast)
SAMN44463305	F7_M_Y	Fermentation_7_After-milling_(Yeast)
SAMN44463306	F7_AM_Y	Fermentation_7_After-mixing_(Yeast)
SAMN44463307	F7_BD_Y	Fermentation_7_Before-distillation_(Yeast)
SAMN44463308	F8_M_Y	Fermentation_8_After-milling_(Yeast)
SAMN44463309	F8_AM_Y	Fermentation_8_After-mixing_(Yeast)
SAMN44463310	F8_BD_Y	Fermentation_8_Before-distillation_(Yeast)
SAMN44463311	F9_M_Y	Fermentation_9_After-milling_(Yeast)
SAMN44463312	F9_AM_Y	Fermentation_9_After-mixing_(Yeast)
SAMN44463313	F9_BD_Y	Fermentation_9_Before-distillation_(Yeast)
SAMN44463314	F10_M_Y	Fermentation_10_After-milling_(Yeast)
SAMN44463315	F10_AM_Y	Fermentation_10_After-mixing_(Yeast)
SAMN44463316	F10_BD_Y	Fermentation_10_Before-distillation_(Yeast)
SAMN44463317	F11_M_Y	Fermentation_11_After-milling_(Yeast)
SAMN44463318	F11_AM_Y	Fermentation_11_After-mixing_(Yeast)
SAMN44463319	F11_BD_Y	Fermentation_11_Before-distillation_(Yeast)
SAMN44463320	F12_M_Y	Fermentation_12_After-milling_(Yeast)
SAMN44463321	F12_AM_Y	Fermentation_12_After-mixing_(Yeast)
SAMN44463322	F12_BD_Y	Fermentation_12_Before-distillation_(Yeast)
SAMN44463323	F13_M_Y	Fermentation_13_After-milling_(Yeast)
SAMN44463324	F13_AM_Y	Fermentation_13_After-mixing_(Yeast)
SAMN44463325	F13_BD_Y	Fermentation_13_Before-distillation_(Yeast)
SAMN44463326	F14_M_Y	Fermentation_14_After-milling_(Yeast)

SAMN44463327	F14_AM_Y	Fermentation_14_After-mixing_(Yeast)
SAMN44463328	F14_BD_Y	Fermentation_14_Before-distillation_(Yeast)
SAMN44463329	F15_M_Y	Fermentation_15_After-milling_(Yeast)
SAMN44463330	F15_AM_Y	Fermentation_15_After-mixing_(Yeast)
SAMN44463331	F15_BD_Y	Fermentation_15_Before-distillation_(Yeast)
SAMN44463332	F1_M_B	Fermentation_1_After-milling_(Bacteria)
SAMN44463333	F1_AM_B	Fermentation_1_After-mixing_(Bacteria)
SAMN44463334	F1_BD_B	Fermentation_1_Before-distillation_(Bacteria)
SAMN44463335	F2_M_B	Fermentation_2_After-milling_(Bacteria)
SAMN44463336	F2_AM_B	Fermentation_2_After-mixing_(Bacteria)
SAMN44463337	F2_BD_B	Fermentation_2_Before-distillation_(Bacteria)
SAMN44463338	F3_M_B	Fermentation_3_After-milling_(Bacteria)
SAMN44463339	F3_AM_B	Fermentation_3_After-mixing_(Bacteria)
SAMN44463340	F3_BD_B	Fermentation_3_Before-distillation_(Bacteria)
SAMN44463341	F4_M_B	Fermentation_4_After-milling_(Bacteria)
SAMN44463342	F4_AM_B	Fermentation_4_After-mixing_(Bacteria)
SAMN44463343	F4_BD_B	Fermentation_4_Before-distillation_(Bacteria)
SAMN44463344	F5_M_B	Fermentation_5_After-milling_(Bacteria)
SAMN44463345	F5_AM_B	Fermentation_5_After-mixing_(Bacteria)
SAMN44463346	F5_BD_B	Fermentation_5_Before-distillation_(Bacteria)
SAMN44463347	F6_M_B	Fermentation_6_After-milling_(Bacteria)
SAMN44463348	F6_AM_B	Fermentation_6_After-mixing_(Bacteria)
SAMN44463349	F6_BD_B	Fermentation_6_Before-distillation_(Bacteria)
SAMN44463350	F7_M_B	Fermentation_7_After-milling_(Bacteria)
SAMN44463351	F7_AM_B	Fermentation_7_After-mixing_(Bacteria)
SAMN44463352	F7_BD_B	Fermentation_7_Before-distillation_(Bacteria)
SAMN44463353	F8_M_B	Fermentation_8_After-milling_(Bacteria)
SAMN44463354	F8_AM_B	Fermentation_8_After-mixing_(Bacteria)
SAMN44463355	F8_BD_B	Fermentation_8_Before-distillation_(Bacteria)
SAMN44463356	F9_M_B	Fermentation_9_After-milling_(Bacteria)
SAMN44463357	F9_AM_B	Fermentation_9_After-mixing_(Bacteria)
SAMN44463358	F9_BD_B	Fermentation_9_Before-distillation_(Bacteria)
SAMN44463359	F10_M_B	Fermentation_10_After-milling_(Bacteria)
SAMN44463360	F10_AM_B	Fermentation_10_After-mixing_(Bacteria)
SAMN44463361	F10_BD_B	Fermentation_10_Before-distillation_(Bacteria)
SAMN44463362	F11_M_B	Fermentation_11_After-milling_(Bacteria)
SAMN44463363	F11_AM_B	Fermentation_11_After-mixing_(Bacteria)
SAMN44463364	F11_BD_B	Fermentation_11_Before-distillation_(Bacteria)
SAMN44463365	F12_M_B	Fermentation_12_After-milling_(Bacteria)
SAMN44463366	F12_AM_B	Fermentation_12_After-mixing_(Bacteria)
SAMN44463367	F12_BD_B	Fermentation_12_Before-distillation_(Bacteria)
SAMN44463368	F13_M_B	Fermentation_13_After-milling_(Bacteria)
SAMN44463369	F13_AM_B	Fermentation_13_After-mixing_(Bacteria)

SAMN44463370	F13_BD_B	Fermentation_13_Before-distillation_(Bacteria)
SAMN44463371	F14_M_B	Fermentation_14_After-milling_(Bacteria)
SAMN44463372	F14_AM_B	Fermentation_14_After-mixing_(Bacteria)
SAMN44463373	F14_BD_B	Fermentation_14_Before-distillation_(Bacteria)
SAMN44463374	F15_M_B	Fermentation_15_After-milling_(Bacteria)
SAMN44463375	F15_AM_B	Fermentation_15_After-mixing_(Bacteria)
SAMN44463376	F15_BD_B	Fermentation_15_Before-distillation_(Bacteria)

Table S3. Read count summary (raw and filtered) for each sample in the metataxonomic analysis.

Sample code	Microbial diversity	Raw Data (number of reads)	Filtered Data (number of reads)
F1_M	Fungal	148642	68947
F1_AM	Fungal	138366	70607
F1_BD	Fungal	153399	61985
F2_M	Fungal	120494	52616
F2_AM	Fungal	131736	57224
F2_BD	Fungal	136971	46875
F3_M	Fungal	154248	78123
F3_AM	Fungal	152430	82505
F3_BD	Fungal	150768	59415
F4_M	Fungal	161898	69562
F4_AM	Fungal	146574	75878
F4_BD	Fungal	121412	121373
F5_M	Fungal	162462	75737
F5_AM	Fungal	137853	69984
F5_BD	Fungal	139518	46507
F6_M	Fungal	117762	48199
F6_AM	Fungal	143696	65033
F6_BD	Fungal	159851	51639
F7_M	Fungal	109210	107095
F7_AM	Fungal	95183	95109
F7_BD	Fungal	28583	28523
F8_M	Fungal	130421	125886
F8_AM	Fungal	140924	140461
F8_BD	Fungal	85352	85168
F9_M	Fungal	103337	102754
F9_AM	Fungal	163228	163111
F9_BD	Fungal	108287	108098
F10_M	Fungal	162851	162664
F10_AM	Fungal	213738	213718
F10_BD	Fungal	161530	161061
F11_M	Fungal	107384	104711
F11_AM	Fungal	109853	109733
F11_BD	Fungal	112430	111059
F12_M	Fungal	117867	117367
F12_AM	Fungal	204398	204338
F12_BD	Fungal	174802	174683
F13_M	Fungal	211540	211409
F13_AM	Fungal	169989	169947
F13_BD	Fungal	186865	186254
F14_M	Fungal	121840	120076

F14_AM	Fungal	219455	219273
F14_BD	Fungal	194795	194690
F15_M	Fungal	214637	214322
F15_AM	Fungal	115489	115471
F15_BD	Fungal	170939	170914
F1_M	Bacterial	108555	107947
F1_AM	Bacterial	118511	118146
F1_BD	Bacterial	116729	115939
F2_M	Bacterial	109024	108391
F2_AM	Bacterial	98752	98137
F2_BD	Bacterial	107854	106430
F3_M	Bacterial	102853	102271
F3_AM	Bacterial	89220	86925
F3_BD	Bacterial	93761	92737
F4_M	Bacterial	100320	99893
F4_AM	Bacterial	98927	98412
F4_BD	Bacterial	114688	112594
F5_M	Bacterial	112704	111710
F5_AM	Bacterial	109535	107935
F5_BD	Bacterial	81869	80443
F6_M	Bacterial	92974	92586
F6_AM	Bacterial	112162	111467
F6_BD	Bacterial	111657	109896
F7_M	Bacterial	141641	136670
F7_AM	Bacterial	114552	110651
F7_BD	Bacterial	116517	107247
F8_M	Bacterial	128458	126371
F8_AM	Bacterial	130492	126925
F8_BD	Bacterial	137076	126579
F9_M	Bacterial	124492	122483
F9_AM	Bacterial	99055	96101
F9_BD	Bacterial	118641	110969
F10_M	Bacterial	107697	105393
F10_AM	Bacterial	96878	94112
F10_BD	Bacterial	105956	97282
F11_M	Bacterial	129772	128356
F11_AM	Bacterial	132292	128403
F11_BD	Bacterial	119571	116204
F12_M	Bacterial	111059	100169
F12_AM	Bacterial	110564	107967
F12_BD	Bacterial	129830	119633
F13_M	Bacterial	124298	123197
F13_AM	Bacterial	113476	111887

F13_BD	Bacterial	129670	127467
F14_M	Bacterial	115232	112618
F14_AM	Bacterial	119209	117847
F14_BD	Bacterial	124879	122632
F15_M	Bacterial	100322	92447
F15_AM	Bacterial	121119	119793
F15_BD	Bacterial	115885	113224

Table S4. Statistical ANOVA analysis based on alpha index and PERMANOVA analysis based on beta index Bray Curtis of fungal metataxonomic analysis. Three groups of analyses are presented: by year, by agave species and by fermentation stage. *P-values* in red represent a statistically significant difference between these conditions at 95% confidence level.

Fungi

Alpha index

Condition		Chao1				Dominance				Shannon			
**Stage**	**Year**	**Average**	**SD**	**CV**	***P-value***	**Average**	**SD**	**CV**	***P-value***	**Average**	**SD**	**CV**	***P-value***
**M**	**2020**	76.50	7.23	9.45%	**0.223**	0.48	0.08	16.95%	**0.013**	2.01	0.29	14.23%	**0.026**
	**2022**	93.11	30.86	33.14%		0.27	0.17	62.29%		2.96	0.88	29.86%	
**AM**	**2020**	63.00	10.14	16.09%	**0.074**	0.61	0.08	13.24%	**0.0003**	1.58	0.29	18.63%	**0.024**
	**2022**	50.00	14.05	28.11%		0.34	0.12	34.50%		2.30	0.64	28.05%	
**BD**	**2020**	64.50	8.12	12.59%	**0.658**	0.52	0.10	20.21%	**0.040**	1.71	0.24	13.97%	**0.053**
	**2022**	62.22	10.34	16.62%		0.38	0.13	33.63%		2.26	0.60	26.56%	
**Stage**	**Agave**	**Average**	**SD**	**CV**	***P-value***	**Average**	**SD**	**CV**	***P-value***	**Average**	**SD**	**CV**	***P-value***
**M**	***Agave angustifolia***	88.44	31.84	36.00%	**0.937**	0.36	0.21	59.92%	**0.8941**	2.66	1.06	39.90%	**0.822**
	Agave americana	82.33	10.60	12.87%		0.37	0.11	30.53%		2.29	0.49	21.57%	
	Agave karwinskii	84.67	14.84	17.53%		0.31	0.08	25.68%		2.64	0.23	8.61%	
**AM**	***Agave angustifolia***	53.11	14.69	27.66%	**0.510**	0.42	0.21	50.97%	**0.6975**	2.15	0.80	37.28%	**0.615**
	Agave americana	64.00	17.35	27.11%		0.51	0.09	17.97%		1.78	0.09	5.21%	
	Agave karwinskii	52.67	6.43	12.21%		0.48	0.07	15.33%		1.82	0.15	8.29%	
**BD**	***Agave angustifolia***	62.78	9.86	15.70%	**0.540**	0.41	0.17	40.44%	**0.7428**	2.16	0.67	31.16%	**0.420**
	Agave americana	59.33	9.86	15.11%		0.49	0.05	9.95%		1.66	0.09	5.47%	
	Agave karwinskii	68.00	8.67	12.74%		0.43	0.08	19.52%		2.06	0.16	7.98%	
**Stage**		**Average**	**SD**	**CV**	***P-value***	**Average**	**SD**	**CV**	***P-value***	**Average**	**SD**	**CV**	***P-value***
**M**		86.47	25.18	29.12%	**0.009**	0.35	0.17	49.06%	**0.2137**	2.58	0.84	32.60%	**0.048**
**AM**		55.20	13.89	25.17%		0.45	0.17	38.32%		2.01	0.63	31.57%	
**BD**		63.13	9.27	14.69%		0.43	0.14	31.25%		2.04	0.55	27.10%	

Beta index

Condition		PERMANOVA		Condition		PERMANOVA		Condition	PERMANOVA	
**Stage**	**Year**	**Pseudo-F**	***P-value***	**Stage**	**Agave**	**Pseudo-F**	***P-value***	**Stage**	**Pseudo-F**	***P-value***
**M**	**2020**	9.93	**0.001**	**M**	***Agave angustifolia***	1.36	**0.215**	**M**	1.05	**0.375**
	**2022**									
**AM**	**2020**	19.38	**0.003**	**AM**	***Agave americana***	1.04	**0.387**	**AM**		
	**2022**									
**BD**	**2020**	9.14	**0.001**	**BD**	***Agave karwinskii***	0.88	**0.530**	**BD**		
	**2022**									

Table S5. Statistical ANOVA analysis based on alpha index and PERMANOVA analysis based on beta index Bray Curtis of bacterial metataxonomic analysis. Three groups of analyses are presented: by year, by agave species and by fermentation stage. *P-values* in red represent a statistically significant difference between these conditions at 95% confidence level.

BACTERIA

Alpha index

Condition		Chao1				Dominance				Shannon			
**Stage**	**Year**	**Average**	**SD**	**CV**	***P-value***	**Average**	**SD**	**CV**	***P-value***	**Average**	**SD**	**CV**	***P-value***
**M**	**2020**	288.08	55.58	19.29%	**0.002**	0.11	0.04	36.13%	**0.266**	4.69	0.46	9.72%	**0.030**
	**2022**	666.66	238.02	35.70%		0.08	0.06	71.32%		5.89	1.14	19.30%	
**AM**	**2020**	239.17	37.51	15.68%	**0.010**	0.05	0.01	18.68%	**0.016**	5.32	0.22	4.15%	**0.038**
	**2022**	430.83	150.94	35.03%		0.16	0.09	59.24%		4.56	0.78	17.10%	
**BD**	**2020**	254.42	76.12	29.92%	**0.005**	0.05	0.01	24.04%	**0.169**	5.34	0.29	5.37%	**0.397**
	**2022**	427.44	109.58	25.64%		0.09	0.06	69.53%		5.10	0.63	12.41%	
**Stage**	**Agave**	**Average**	**SD**	**CV**	***P-value***	**Average**	**SD**	**CV**	***P-value***	**Average**	**SD**	**CV**	***P-value***
**M**	***Agave angustifolia***	603.36	305.39	50.62%	**0.309**	0.06	0.04	54.70%	**0.037**	5.92	1.10	18.49%	**0.073**
	Agave americana	389.93	157.37	40.35%		0.12	0.05	43.00%		4.65	0.73	15.65%	
	Agave karwinskii	376.13	83.47	22.19%		0.13	0.05	37.44%		4.65	0.21	4.41%	
**AM**	***Agave angustifolia***	353.84	171.92	48.59%	**0.956**	0.14	0.10	71.59%	**0.253**	4.60	0.77	16.75%	**0.170**
	Agave americana	374.63	190.09	50.74%		0.05	0.01	27.41%		5.48	0.49	8.99%	
	Agave karwinskii	334.67	72.23	21.58%		0.09	0.03	36.16%		5.02	0.24	4.86%	
**BD**	***Agave angustifolia***	404.99	143.52	35.43%	**0.235**	0.09	0.06	66.81%	**0.311**	5.08	0.63	12.32%	**0.570**
	Agave americana	298.87	7.59	2.54%		0.05	0.01	26.60%		5.42	0.29	5.37%	
	Agave karwinskii	277.33	94.93	34.23%		0.05	0.01	22.61%		5.35	0.31	5.79%	
**Stage**		**Average**	**SD**	**CV**	***P-value***	**Average**	**SD**	**CV**	***P-value***	**Average**	**SD**	**CV**	***P-value***
**M**		515.23	265.20	51.47%	**0.041**	0.09	0.05	56.06%	**0.299**	5.41	1.09	20.12%	**0.180**
**AM**		354.17	151.55	42.79%		0.11	0.09	77.88%		4.86	0.72	14.75%	
**BD**		358.23	128.95	36.00%		0.08	0.05	69.08%		5.20	0.52	10.06%	

Beta index

Condition		PERMANOVA		Condition		PERMANOVA		Condition	PERMANOVA	
**Stage**	**Year**	**Pseudo-F**	***P-value***	**Stage**	**Agave**	**Pseudo-F**	***P-value***	**Stage**	**Pseudo-F**	***P-value***
**M**	**2020**	8.54	**0.001**	**M**	***Agave angustifolia***	1.49	**0.153**	**M**	4.20	**0.001**
	**2022**									
**AM**	**2020**	5.95	**0.001**	**AM**	***Agave americana***	1.68	**0.103**	**AM**		
	**2022**									
**BD**	**2020**	6.47	**0.001**	**BD**	***Agave karwinskii***	2.06	**0.029**	**BD**		
	**2022**									
